# The Role of [^68^Ga]Ga-DOTA-SSTR PET Radiotracers in Brain Tumors: A Systematic Review of the Literature and Ongoing Clinical Trials

**DOI:** 10.3390/cancers14122925

**Published:** 2022-06-14

**Authors:** Paolo Palmisciano, Gina Watanabe, Andie Conching, Christian Ogasawara, Gianluca Ferini, Othman Bin-Alamer, Ali S. Haider, Maria Gabriella Sabini, Giacomo Cuttone, Sebastiano Cosentino, Massimo Ippolito, Giuseppe E. Umana

**Affiliations:** 1 Department of Neurosurgery, University of Cincinnati College of Medicine, Cincinnati, OH 45267, USA; palmispo@ucmail.uc.edu; 2 John A. Burns School of Medicine, University of Hawai’i, Honolulu, HI 96822, USA; ginaw@hawaii.edu (G.W.); andiec@hawaii.edu (A.C.); cogasawa@hawaii.edu (C.O.); 3 Department of Radiation Oncology, REM Radioterapia SRL, 95125 Viagrande, Italy; gianluca.ferini@grupposamed.com; 4 Department of Neurosurgery, University of Pittsburgh Medical Center, Pittsburgh, PA 15232, USA; binalameroa@upmc.edu; 5 Department of Neurosurgery, The University of Texas M.D. Anderson Cancer Center, Houston, TX 77030, USA; aalam@mdanderson.org; 6 Department of Medical Physics, Cannizzaro Hospital, 95021 Catania, Italy; mgabsabini@gmail.com; 7 National Laboratory of South, National Institute for Nuclear Physics (LNS-INFN), 95125 Catania, Italy; cuttone@lns.infn.it; 8 Department of Advanced Technologies, Nuclear Medicine and PET, Cannizzaro Hospital, 95021 Catania, Italy; sebastiano.cosentino@aoec.it (S.C.); massimo.ippolito@aoec.it (M.I.); 9 Department of Neurosurgery, Trauma Center, Gamma Knife Center, Cannizzaro Hospital, 95021 Catania, Italy

**Keywords:** Gallium PET, meningioma, neuro-oncology, nuclear medicine, PET, theranostics

## Abstract

**Simple Summary:**

[^68^Ga]Ga-DOTA-SSTR PET imaging has recently been introduced in the management of patients with brain tumors, mostly meningiomas and pituitary adenomas or carcinomas. The current literature demonstrated the superior diagnostic accuracy of this imaging modality, especially for lesions difficult to be detected or characterized on conventional imaging protocols, such as skull base or transosseous meningiomas. [^68^Ga]Ga-DOTA-SSTR PET tracers also seem to provide superior volume contouring for radiotherapy planning and may also be used to evaluate the tumor’s overexpression of somatostatin receptors for devising patient-tailored peptide receptor radionuclide therapy. In this review, we comprehensively analyzed the current literature discussing the implementation of [^68^Ga]Ga-DOTA-SSTR PET imaging in brain tumors, further presenting ongoing clinical trials and suggesting potential future applications.

**Abstract:**

Background: The development of [^68^Ga]Ga-DOTA-SSTR PET tracers has garnered interest in neuro-oncology, to increase accuracy in diagnostic, radiation planning, and neurotheranostics protocols. We systematically reviewed the literature on the current uses of [^68^Ga]Ga-DOTA-SSTR PET in brain tumors. Methods: PubMed, Scopus, Web of Science, and Cochrane were searched in accordance with the PRISMA guidelines to include published studies and ongoing trials utilizing [^68^Ga]Ga-DOTA-SSTR PET in patients with brain tumors. Results: We included 63 published studies comprising 1030 patients with 1277 lesions, and 4 ongoing trials. [^68^Ga]Ga-DOTA-SSTR PET was mostly used for diagnostic purposes (62.5%), followed by treatment planning (32.7%), and neurotheranostics (4.8%). Most lesions were meningiomas (93.6%), followed by pituitary adenomas (2.8%), and the DOTATOC tracer (53.2%) was used more frequently than DOTATATE (39.1%) and DOTANOC (5.7%), except for diagnostic purposes (DOTATATE 51.1%). [^68^Ga]Ga-DOTA-SSTR PET studies were mostly required to confirm the diagnosis of meningiomas (owing to their high SSTR2 expression and tracer uptake) or evaluate their extent of bone invasion, and improve volume contouring for better radiotherapy planning. Some studies reported the uncommon occurrence of SSTR2-positive brain pathology challenging the diagnostic accuracy of [^68^Ga]Ga-DOTA-SSTR PET for meningiomas. Pre-treatment assessment of tracer uptake rates has been used to confirm patient eligibility (high somatostatin receptor-2 expression) for peptide receptor radionuclide therapy (PRRT) (i.e., neurotheranostics) for recurrent meningiomas and pituitary carcinomas. Conclusion: [^68^Ga]Ga-DOTA-SSTR PET studies may revolutionize the routine neuro-oncology practice, especially in meningiomas, by improving diagnostic accuracy, delineation of radiotherapy targets, and patient eligibility for radionuclide therapies.

## 1. Introduction

Current imaging modalities for brain tumor diagnoses mainly comprise CT and/or MRI scans, which confer favorable sensitivity and specificity for outlining initial suspects of lesions [1,2]. However, CT and MRI scans may be insufficient to provide a detailed characterization of intracranial masses, in some cases requiring advanced imaging techniques for enhancing the differential diagnosis and supporting the pre-treatment planning of optimal therapeutic options. The increased advances and availability of positron emission tomography/computed tomography (PET/CT) imaging in oncology and neuro-oncology practices have encouraged the research and development of multiple PET radiopharmaceuticals for diagnostic and treatment purposes [1,2]. The use of radiolabeled amino acid PET tracers, which bind to specific tumor-expressed receptors, offers improved accuracy in defining the tumor-to-background contrast and in tailoring treatments [3]. More recently, Gallium-68 (^68^Ga) has attracted a lot of interest as an alternative positron emitter to the most common ^18^F-2-fluoro-2-deoxy-D-glucose (^18^F-FDG) [4]. ^68^Ga proved to be a versatile tool in several oncology and non-oncology applications, providing a short imaging time and cost-effective cyclotron-free production [5]. In particular, ^68^Ga-labeling of DOTA chelator-conjugated somatostatin analogs allows to detect and bind with high-affinity tumors expressing selected somatostatin receptors [6]. Among the different [^68^Ga]Ga-DOTA tracers, [^68^Ga]Ga-DOTA-D-Phe1-Tyr3-octreotide ([^68^Ga]Ga-DOTATOC), [^68^Ga]Ga-DOTA-D-Phe1-Tyr3-octreotate ([^68^Ga]Ga-DOTATATE), and [^68^Ga]Ga-DOTA-D-Phe1-Nal3-octreotide ([^68^Ga]Ga-DOTANOC) represent three of the most common agents currently used in PET imaging of neuroendocrine and central nervous system (CNS) tumors [7,8].

In neuro-oncology, [^68^Ga]Ga-DOTA-SSTR PET tracers have been mostly studied for meningiomas and pituitary adenomas [7,9]. Both pathologies show overexpression of somatostatin subtype receptors 2 (SSTR2), to which [^68^Ga]Ga-DOTA-chelator PET tracers are able to bind with strong affinity [10,11,12]. PET/CT and, more recently, hybrid PET/MRI systems, show a superior diagnostic accuracy compared to CT and MRI protocols, allowing a better detection and volume definition of skull base and transosseous meningiomas, and also post-surgery residual tumors [13,14,15,16]. With these premises, [^68^Ga]Ga-DOTA-SSTR PET scans are frequently implemented as imaging adjuncts to guide surgical resection and radiation treatment planning of complex meningiomas, improving safe and effective target delineation [17,18,19,20]. [^68^Ga]Ga-DOTA-SSTR PET scans may also be used to study the pretherapeutic tumor uptake of DOTA-chelator tracers and predict tumor response to targeted DOTA-labeled β-emitting radionuclides (i.e., neurotheranostics), as described for radiation-resistant meningiomas [21].

In this systematic review, we comprehensively summarized the various applications of [^68^Ga]Ga-DOTA-SSTR PET tracers in neuro-oncology practice, primarily focusing on their implementation in diagnostics, treatment planning, and neurotheranostics settings.

## 2. Materials and Methods

### 2.1. Literature Search

A systematic review was conducted in accordance with the Preferred Reporting Items for Systematic Reviews and Meta-Analyses (PRISMA) guidelines [22] and registered to PROSPERO (CRD42022325392). PubMed, Scopus, Web of Science, and Cochrane were searched from database inception to 7 April 2022, using the exact search query: [(Gallium-68 DOTATOC PET OR 68 GA-DOTATOC OR Gallium-68 DOTATATE OR 68GA-DOTATATE OR Gallium-68 DOTANOC OR 68GA-DOTANOC) AND (tumor OR oncology OR neoplasm)]. Articles were uploaded to Mendeley, and duplicates were deleted. ClinicalTrial.gov was then searched in the same fashion to identify ongoing clinical trials evaluating the use of [^68^Ga]Ga-DOTA-SSTR PET studies in patients with brain tumors.

### 2.2. Study Selection

A priori inclusion and exclusion criteria were defined. Articles written in English were included if they described the use of [^68^Ga]Ga-DOTA-SSTR PET imaging in patients with brain tumors for: (1) diagnostics purposes, (2) treatment planning, (3) eligibility for therapy with DOTA-tracers β-emitting radionuclides (i.e., neurotheranostics). Articles were excluded if they: (1) were literature reviews, cadaver studies, animal studies, or study protocols, (2) described different uses of [^68^Ga]Ga-DOTA-SSTR PET imaging not for brain tumors, (3) reported the use of different molecular nuclear medicine imaging techniques. [^68^Ga]Ga-DOTA-SSTR PET imaging defined the use of [^68^Ga]Ga-DOTA-chelator PET studies specifically targeting tissues and/or lesions expressing somatostatin receptors (SSTR).

Two independent authors (G.W. and C.O.) screened titles and abstracts of all collected articles, and then assessed full texts of studies that met inclusion criteria. Disagreements were settled by a third author (P.P.). Eligible articles were included upon the pre-specified criteria and references were searched to retrieve additional articles.

### 2.3. Data Extraction

Two independent reviewers (G.W. and C.O.) extracted data, which were then confirmed by an additional reviewer (P.P.). Missing data were not reported by the authors. Extracted data included: authors, year, the reason for the use of [^68^Ga]Ga-DOTA-SSTR PET studies, sample size, number of lesions, pathology and location, tracer and administered dose, SUV max, clinico-radiological findings.

### 2.4. Data Synthesis and Quality Assessment

The primary outcomes of interest were current applications of [^68^Ga]Ga-DOTA-SSTR PET studies in patients with brain tumors and their impact on clinical practice. For each article, the level of evidence was appraised using the 2011 Oxford Centre For Evidence-Based Medicine guidelines [23]. A meta-analysis was precluded because all included studies had level IV evidence and hazard ratios could not be deducted. The risk of bias in each article was independently assessed by two authors (P.P. and G.F.) using the Joanna Briggs Institute checklists for case reports and case series [24,25]. Continuous variables are summarized as medians or means and ranges, while categorical variables are reported as frequencies and percentages.

## 3. Results

### 3.1. Study Selection

**Figure 1:**
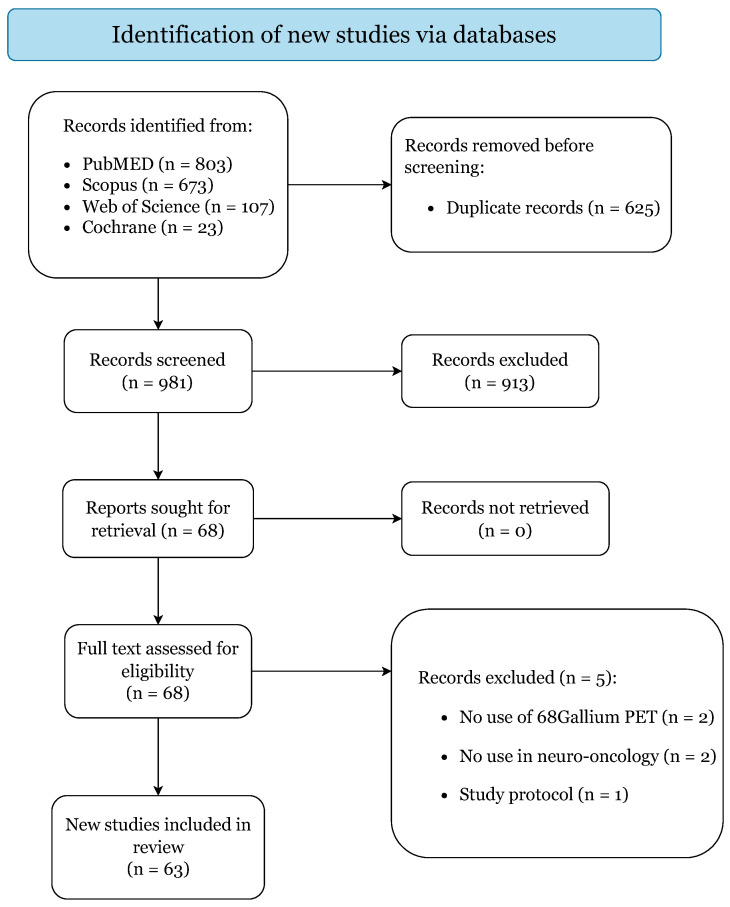
PRISMA 2020 flow diagram.

Figure 1 illustrates the literature search and study selection process. The initial search yielded 1606 published articles (PubMed: 803; Scopus: 673; Web of Science: 107; Cochrane: 23). A total of 63 studies were included with the a priori criteria: 25 were case reports and 38 were case series, categorized with levels of evidence IV and V, respectively [13,14,15,16,17,18,19,20,21,26,27,28,29,30,31,32,33,34,35,36,37,38,39,40,41,42,43,44,45,46,47,48,49,50,51,52,53,54,55,56,57,58,59,60,61,62,63,64,65,66,67,68,69,70,71,72,73,74,75,76,77,78,79]. The second search returned 116 clinical trials, of which 4 ongoing trials were included [80,81,82,83]. A quality assessment resulted in a low risk of bias for all included articles, predisposing this review to an overall low risk of bias (Supplementary File S1).

### 3.2. Overview of [^68^Ga]Ga-DOTA-SSTR PET Studies in Neuro-Oncology

Table 1 summarizes and separates current applications of [^68^Ga]Ga-DOTA-SSTR PET in patients with brain tumors. A total of 1030 patients with 1277 lesions were included. [^68^Ga]Ga-DOTA-SSTR PET studies were mostly implemented for diagnostic purposes in 644 patients (62.5%) with 867 lesions (67.9%), followed by radiotherapy/surgery planning in 337 patients (32.7%) with 356 lesions (27.9%), and by assessment of somatostatin receptor-2 (SSTR2) expression for peptide receptor radionuclide therapy (PRRT) (i.e., neurotheranostics) in 49 patients (4.8%) with 54 lesions (4.2%). Meningioma (total: 1196 lesions, 93.6%) comprised the most common pathology in all groups, followed by pituitary adenomas (total: 36 lesions, 2.8%). DOTATOC was the most frequent tracer used overall (total: 548 patients, 53.2%), but DOTATATE was used more in patients undergoing diagnostic [^68^Ga]Ga-DOTA-SSTR PET studies (329, 51.1%). Purandare et al. [54] described the use of DOTANOC tracer in 40 patients to differentiate meningiomas from dural-based brain metastases, and Pelak et al. [69] to delineate tumor volumes for proton therapy in 19 patients with grade-1 meningiomas. Seysthal et al. [21] lacked a clear distinction in the use of DOTATOC or DOTATATE, grouping all 20 of their patients in a single DOTATOC/DOTATATE group.

### 3.3. [^68^Ga]Ga-DOTA-SSTR PET Diagnostic Studies

Table 2 overviews all studies using [^68^Ga]Ga-DOTA-SSTR PET for diagnostic purposes in 644 patients with 867 lesions. Most lesions were meningiomas (93%), followed by pituitary adenomas (4.2%). Less common SSTR2-positive brain tumors were also described, including: brain metastases (15 patients, 1.7%) from breast cancer (6 patients) [54,55,64], neuroendocrine neoplasms (NEN) (2 patients) [70,76], gastric cancer (1 patient) [40], head and neck cancer (1 patient) [54], lymphoma (1 patient) [54], rectal cancer (1 patient) [54], renal cell carcinoma (1 patient) [54], thyroid cancer (1 patient) [48], and uterine cancer (1 patient) [54]; pituitary carcinomas (3 patients, 0.2%) [47,67,75]; granulomatous inflammation (2 patients, 0.2%) [40,42]; lymphoma (2 patients, 0.2%) [40,58]; glomus jugulare (1 patient, 0.1%) [73]; hemangioma (1 patient, 0.1%) [78]; and schwannoma (1 patient, 0.1%) [74]. DOTATATE (54.5%) was utilized more frequently than DOTATOC (45.5%). In meningiomas, [^68^Ga]Ga-DOTA-SSTR PET studies were implemented to: (1) evaluate intraosseous or infracranial extension [15,31,79]; (2) detect lesions difficult-to-identify on MRI [30,63]; (3) differentiate residual/recurrent tumor tissue versus non-tumor tissue [14], such as surgical scarring [38] or post-therapy changes [53]; (4) confirm the diagnosis of meningiomas (high tracer uptake) [35,71,72] and differentiate those from other pathologies with lower tracer uptakes, both tumoral [44,54,55,58] and non-tumoral [40,42]; (5) stage recurrent or systemic disease [34,50,56]; (6) quantify tracer uptake (SSTR2 expression) [61,62,77] and match/mismatch with FDG uptake (metabolic rate) [35,52]; (7) evaluate postoperative extent of resection [60,65]; (8) predict tumor growth [45]; and appraise the diagnostic accuracy of hybrid PET/MRI scans [13,37,63]. In pituitary adenomas, [^68^Ga]Ga-DOTA-SSTR PET studies were utilized to confirm biologically active post-surgical residual tumor tissue in concomitance with FDG PET/CT studies [16], or for differential diagnosis with synchronous meningiomas [44]. ^68^Ga-DOTATATE PET was used in the three patients with pituitary carcinomas to detect systemic metastases for disease staging [47,67,75].

### 3.4. [^68^Ga]Ga-DOTA-SSTR PET for Planning Radiotherapy Protocols and/or Surgical Resection

Table 3 reports all studies utilizing [^68^Ga]Ga-DOTA-SSTR PET for planning radiotherapy protocols and/or surgical resection in 337 patients with 356 lesions. All lesions were meningiomas (99.7%), except for one pituitary carcinoma reported by d’Amico et al. [36]. DOTATOC (78.2%) was utilized more frequently than DOTATATE (21.8%), and Pelak et al. [69] also reported the use of DOTANOC in 19 patients. [^68^Ga]Ga-DOTA-SSTR PET studies were mostly fused with CT/MRI studies and implemented to improve volume contouring for radiation planning, such as stereotactic radiotherapy (SRT) [20,26,28,33], intensity-modulated radiotherapy (IMRT) [27,29], conventional radiotherapy [46,49,51,66], cyber knife radiosurgery (CKRS) [17,18], and carbon/proton therapy [32,69]. The implementation of [^68^Ga]Ga-DOTA-SSTR PET proved to significantly improve target definition in meningiomas of the skull base, parafalcine, intraosseous, or affecting the optic nerve sheath, also favoring reduced radiation doses to adjacent organs at risk. Guinto-Nishimura et al. [19] registered and uploaded [^68^Ga]Ga-DOTA-SSTR studies to their neuronavigation system for intraoperative PET-guided meningioma resection. D’Amico et al. [36] reported improved target definition and contouring of a residual pituitary carcinoma infiltrating the right cavernous sinus.

### 3.5. [^68^Ga]Ga-DOTA-SSTR PET for Planning Neurotheranostics Therapy

Table 4 presents all studies using [^68^Ga]Ga-DOTA-SSTR PET for planning neurotheranostics therapy in 49 patients with 54 lesions. Most lesions were meningiomas (74.1%) followed by high-grade gliomas (22.2%) and pituitary carcinomas (3.7%). DOTATOC was used in 23 patients (46.9%), DOTATATE in 6 patients (12.2%), and unclear use of DOTATOC or DOTATATE was described in 20 patients (40.8%). ^68^Ga-DOTATATE PET studies were mostly utilized for assessing SSTR2 expression and eligibility for [^177^Lu]Lu-DOTATATE therapy in both recurrent meningiomas [21] and pituitary carcinomas [41,68]. Collamati et al. [39] devised a pilot study on patients with meningiomas or high-grade gliomas to quantify [^68^Ga]Ga-DOTATOC uptake by targeted tumors and estimate [^90^Y]Y-DOTATOC uptake, planning to develop a specific probe used for intraoperative radiotracer-guided tumor resection. Verburg et al. [57] evaluated selective intraarterial injection of [^68^Ga]Ga-DOTATATE in meningiomas and compared tracer uptake rates to those following venous [^68^Ga]Ga-DOTATATE infusion, with the goal of better evaluating eligibility for [^177^Lu]Lu-DOTATATE therapy in patients with insufficient tracer uptake after venous [^68^Ga]Ga-DOTATATE infusion.

### 3.6. Ongoing Clinical Trials Investigating [^68^Ga]Ga-DOTA-SSTR PET in Neuro-Oncology

Four clinical trials are currently ongoing—three interventional [80,82,83] and one observational [81] (Table 5). The observational trial led by Ivanidze [81] is expected to evaluate the diagnostic accuracy of hybrid [^68^Ga]Ga-DOTATATE PET/MRI in meningiomas and other SSTR2-positive brain tumors compared to MRI alone, and secondarily correlate rates of tracer uptake to the expressions of SSTR2, Ki67, progesterone receptor, and EGFR. The interventional trial led by Johnson [82] is focused on appraising the diagnostic accuracy of [^68^Ga]Ga-DOTATATE PET/CT in residual meningiomas and measuring their metabolic response to radiotherapy. The interventional trial led by Merrell [83] proposes the use of [^68^Ga]Ga-DOTATATE PET/MRI to assess meningioma eligibility for [^177^Lu]Lu-DOTATATE therapy and simultaneously assess rates of progression-free survival, overall survival, and adverse events. Filipsson Nyström [80] is currently conducting the only interventional trial in patients with pituitary adenomas to quantify and compare rates of [^68^Ga]Ga-DOTATOC uptake between tumors and normal pituitary tissue, and secondarily to correlate tracer uptake rates to SSTR2 expression, to report adverse events, and to detect post-surgery tumor recurrences.

## 4. Discussion

A growing body of literature is currently focusing on analyzing the role of [^68^Ga]Ga-DOTA-SSTR PET studies in neuro-oncology, which have proved to be effective and safe for diagnostic and treatment planning purposes. However, the high variability in applications, SSTR2-positive diseases, and findings may pose some challenges in defining the pros and cons of their implementation in routine practice. In this review, we aimed to provide a comprehensive summary of the current literature reporting the use of [^68^Ga]Ga-DOTA-SSTR PET for brain tumors, hoping to assist all physicians involved in the multidisciplinary management of neuro-oncology patients.

### 4.1. PET Imaging in Neuro-Oncology: [^68^Ga]Ga-DOTA-SSTR Radiotracers

**Figure 2:**
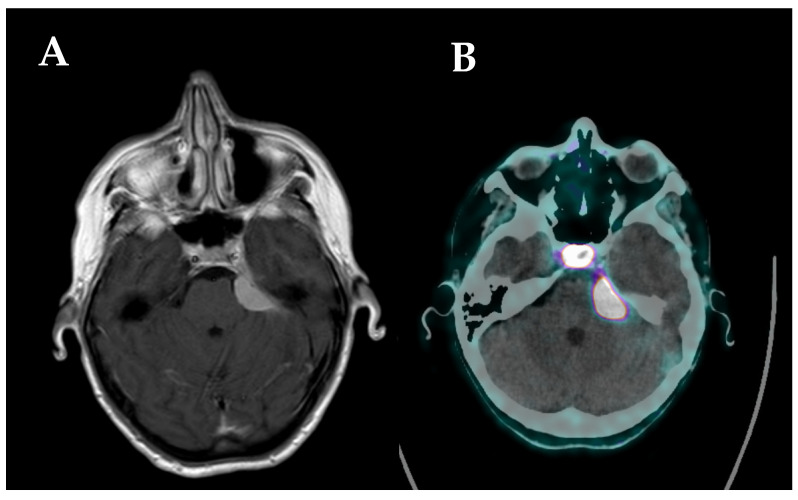
(**A**) MRI scan showing a left sphenopetrosal lesion suspected to be meningioma; (**B**) [^68^Ga]Ga-DOTATOC PET/CT scan showing high tracer uptake of the left sphenopetrosal lesion, suggesting the diagnosis of meningioma, normal tracer uptake of the pituitary gland.

Although multiparametric MRI represents the current imaging gold standard in primary and metastatic brain neoplasms, PET studies have the unique and complementary ability to evaluate and characterize the metabolic patterns within the tumor and non-tumor tissues through the use of selected radiolabeled tracers [84,85,86]. The roles of different PET tracers in tumor diagnosis and post-treatment response assessment have been largely discussed and validated by international consensuses and recommendations [87,88,89,90]. [^18^F]F-FDG and amino acid tracers (i.e., [^11^C]C-MET, [^18^F]F-FET, and [^18^F]F-FDOPA) are mostly used in patients with gliomas, brain metastases, and primary central nervous system lymphomas (PCNSL). They show variable sensitivity and specificity in differentiating tumor tissue from the normal brain tissue, distinguishing post-treatment changes from tumor recurrences, and, more recently, predicting molecular patterns and patient prognosis when implemented for radiomics analyses [91,92]. In view of meningiomas’ overexpression of SSTR, especially SSTR2, [^68^Ga]Ga-labeled DOTA (i.e., somatostatin analogue) tracers targeting SSTR, primarily developed for neuroendocrine tumors, have been largely used to allow highly selected meningioma uptake, low healthy brain tissue uptake, and, thus, higher specificity in the tumor-to-background contrast with excellent tumor visualization [59,79]. To date, three radiotracers have been implemented in neuro-oncology: (1) DOTATATE, targeting SSTR2; (2) DOTATOC, targeting SSTR2 and SSTR5; and (3) DOTANOC targeting SSTR2, SSTR3, and SSTR5. Their main drawback pertains to ^68^Ga’s short half-life (68 min), which makes necessary the availability of highly expensive ^68^Ge/^68^Ga generators in-house or within easy reach. Yet, the lack of patient preparation, the easy tracer synthesis, and the superior diagnostic accuracy compared to other radiotracers, make [^68^Ga]Ga-DOTA-SSTR PET the preferred modality in SSTR-positive tumors, including meningiomas and pituitary neoplasms (Figure 2). In addition, newer hybrid PET/MRI systems have further improved the diagnostic performances of PET studies for brain tumors, combining PET high accuracy with MRI high morphological tumor visualization [13].

### 4.2. Diagnostic [^68^Ga]Ga-DOTA-SSTR PET Studies

**Figure 3:**
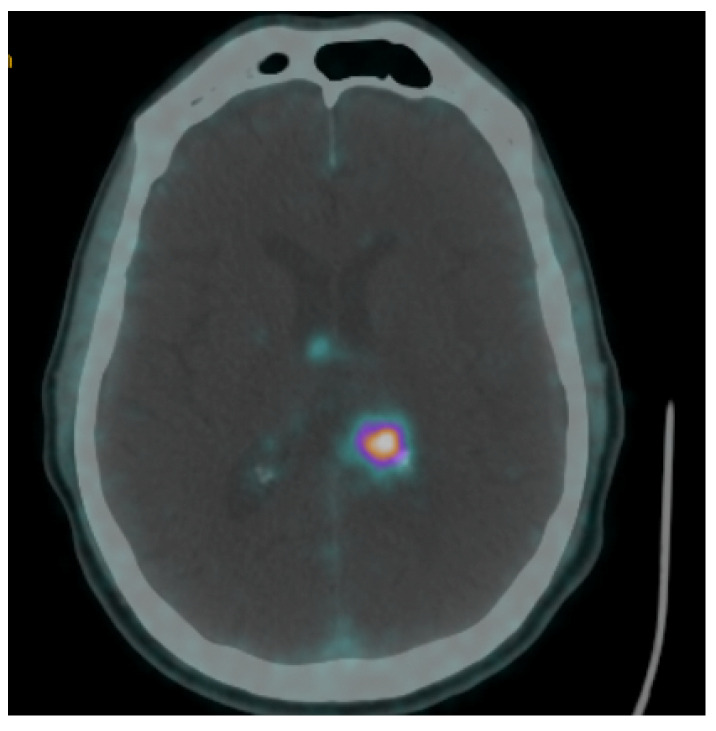
[^68^Ga]Ga-DOTATOC PET/CT scan showing a left intraventricular lesion with high tracer uptake in a patient with a history of carcinoid. GK treatment was planned by contouring the margins of the lesion with tracer uptake.

Since the study by Henze et al. [11] in 2001, [^68^Ga]Ga-DOTA-SSTR PET studies have expanded beyond neuroendocrine neoplasms in neuro-oncology, primarily for meningiomas, with the goal to improve tumor detection, differentiation, and extent-of-infiltration. While most meningiomas are easily identifiable at standard MRIs by showing typical pathognomonic features, lesions with intraosseous extensions, invading the skull base, or adjacent to the falx cerebri, may pose some diagnostic challenges [63,93,94,95]. By selectively binding to SSTR, [^68^Ga]Ga-DOTA-SSTR radiotracers allow for targeted uptake by SSTR-positive neoplasms and high tumor-to-background contrast, offering higher accuracy in evaluating the infracranial/transosseous extent of meningiomas infiltration and detection of synchronous lesions undetected by MRI studies [31,34]. This also provides improved accuracy in preoperatively differentiating suspected meningiomas from different lesions. As confirmed by Purandare et al. [54] and Unterrainer et al. [55], [^68^Ga]Ga-DOTA-SSTR PET can be effectively used to differentiate meningiomas against dural-based brain metastases, also when synchronous in the same patient, despite their largely similar imaging patterns at standard MRI studies. Similarly, Klingenstein et al. [40], Vay et al. [71], and Yarmohammadi et al. [72] proved that [^68^Ga]Ga-DOTA-SSTR PET may distinguish optic nerve sheath meningiomas from other non-tumor optic pathway lesions with higher accuracy than other imaging studies, allowing for prompt surgical/radiation planning on a case-by-case basis. However, in some cases, tumors may mimic meningiomas by presenting unexpected high tracer uptake, including PCNSLs [58] or brain metastases [64] (Figure 3). Hence, despite the promising results, [^68^Ga]Ga-DOTA-SSTR PET should still be considered only as a valuable diagnostic adjunct complementary to preoperative MRI, suitable to aid the multidisciplinary management of neuro-oncological patients but required to be confirmed by histopathology reports.

Rachinger et al. [14] defined a diagnostic threshold of 2.3 for SUVmax to discriminate meningiomas from tumor-free tissue in both pre-treatment and post-treatment settings. Although their limited cohort (*n* = 21) appears insufficient to universally prove their findings, which would require further external validation with larger studies, their threshold has largely been used in the literature to differentiate residual or recurrent meningiomas from post-treatment changes, such as radiation necrosis, scarring, and pseudoprogression [38,53]. Similarly, post-operative [^68^Ga]Ga-DOTA-SSTR PET has also been studied to quantify the extent of meningioma resection in comparison to postoperative MRI and intraoperative Simpson grading, showing lower rates of false-negative and superior detection of tumor remnants [65]. From these findings, a newer “Copenhagen grading” system, including postoperative [^68^Ga]Ga-DOTATOC PET and biopsy confirmation of peri-cavitary “areas of doubt”, has been preliminarily proposed to evaluate the completeness of meningioma resection.

The diagnostic role of [^68^Ga]Ga-DOTA-SSTR PET studies has also been investigated in pituitary adenomas and carcinomas, in view of their SSTR overexpression. As the normal pituitary gland is characterized by physiological tracer uptake, Zhao et al. [16] showed that combined ^18^F-FDG and [^68^Ga]Ga-DOTA-SSTR PET studies may assist in postoperatively differentiate residual adenomas (high FDG uptake, low DOTA uptake) from the remaining functioning pituitary tissue (low FDG uptake, high DOTA uptake). In contrast, pituitary carcinomas present higher tracer uptake compared to the normal pituitary gland, with previous studies reporting high diagnostic accuracy of [^68^Ga]Ga-DOTA-SSTR PET in confirming the diagnosis and enabling the detection of concurrent systemic metastases [43,47,67].

### 4.3. [^68^Ga]Ga-DOTA-SSTR PET for Planning Radiotherapy Protocols and/or Surgical Resection

Accurate tumor volume contouring and target definition are of vital importance for optimizing the planning of surgical resection and radiotherapy protocols. This is especially true in recurrent tumors, such as malignant meningiomas or pituitary carcinomas, characterized by aggressive invasive growth patterns, which often require high doses of radiation, and by post-treatment changes, which may pose some challenges in the delineation of target volumes using morphological imaging studies [96,97,98,99]. In addition, skull base meningiomas are frequently difficult to contour at contrast-MRI and/or CT, as the degree of bone/dura infiltration may be underestimated. As discussed above, PET elicits high tumor-to-background ratios by allowing the detection of receptor overexpression beyond the morphological extent of conventional imaging studies, offering a superior tumor visualization and target definition [100]. The use of [^68^Ga]Ga-DOTA-SSTR PET for treatment planning has been first described by Milker-Zabel et al. [26], who imported and fused CT, MRI, and PET studies in their planning software for fractionated stereotactic radiotherapy (FSRT). The authors compared planning target volumes (PTVs) on fused PET to those on CT/MRI only, reporting improved PTV delineation in 73% of patients after PET-fused planning, which better identified the transosseous extent of meningiomas. Comparable results were also obtained in other studies, which further reported [^68^Ga]Ga-DOTA-SSTR PET detection of additional information on meningiomas not detected at MRI [28,69] and superior target delineation of post-surgery/radiation meningioma recurrence from post-treatment tissue scarring or edema/pseudoprogression [27,29]. The role of [^68^Ga]Ga-DOTA-SSTR PET for tumor volume contouring has been validated for FSRT [20,66], radiosurgery [17,18], and proton/carbon therapy [32,69] planning, optimizing PTV delineation, improving target dose escalation, and minimizing radiation to organs at risk, especially for the highly challenging optic nerve sheath meningiomas [18]. Likewise, d’Amico et al. [36] confirmed the potential benefits of using [^68^Ga]Ga-DOTA-SSTR PET for radiosurgery planning in pituitary carcinomas infiltrating the cavernous sinus, as it allows precise tumor contouring of residual post-surgery volumes.

In contrast to the well-described PET-guided glioma surgery [101], the use of intraoperative [^68^Ga]Ga-DOTA-SSTR PET for navigation-guided meningioma resection has been less investigated. To date, only Guinto-Nishimura et al. [19] reported their experience with [^68^Ga]Ga-DOTATOC PET-guided resection of one primary intraosseous meningioma. In this technical note, the authors noted their ability to achieve gross total tumor removal by including in the resection peripheral bone areas showing high tracer uptake, which appeared macroscopically intact and extended beyond the tumor margins identified on the MRI. The postoperative pathology report confirmed the presence of tumor cells in the PET-positive peripheral bone and their absence in the specimen’s surgically resected margins. In addition, the fusion of PET/CT with MRI images, coupled with their integration with the navigation system, allowed for high accuracy for intraoperatively visualizing radiological anatomical structures and tumor margins, without altering the surgeon’s performance, compared to routine navigation-guided surgical protocols. Hence, [^68^Ga]Ga-DOTA-SSTR PET-guided meningioma resection may be feasible and effective in challenging cases with great intraosseous and/or skull base extension, but further surgical studies should be conducted to analyze the surgical performances from multiple centers and operators.

### 4.4. [^68^Ga]Ga-DOTA-SSTR PET for Planning Neurotheranostics Therapy

Precision medicine approaches are constantly expanding in neuro-oncology to devise patient-tailored treatments directed against individual molecular and genetic profiles, responsible for the high intra-tumor and between-tumor heterogeneity, so as to selectively target cancer cells while minimizing damage to the healthy brain tissue [102]. The development of neurotheranostics in nuclear oncology follows the same path, identifying “theranostic pairs” composed of one diagnostic and one therapeutic nucleotide with identical target molecules [103]. At first, the diagnostic radiotracer selectively binds to specific target receptors to identify the tumor’s expression and molecular pathology. Secondarily, the therapeutic radionuclide is paired with the same tumor-specific biomarker and administered to deliver a dose-effective and selective radioablative dose only to the tumor tissue. Among the literature on [^68^Ga]Ga-DOTA-based theranostic agents in neuro-oncology, the only validated theranostic pair is composed of [^68^Ga]Ga-DOTATATE (diagnostic) and [^177^Lu]Lu-DOTATATE (therapeutic), clinically used for meningiomas [21] or pituitary carcinomas [41,68]. Recent studies confirmed that higher SSTR2 expression by targeted tumors (evidence with higher uptake of [^68^Ga]Ga-DOTATATE) predicts longer and more favorable treatment responses [21,41,68]. Verburg et al. [57] also demonstrated that selective transfemoral intraarterial injection of DOTATATE significantly increased tracer uptake from meningiomas, which showed insufficient tracer uptake after standard venous infusion. This technique, which proved to be well tolerated and without any risk of complications, may be further implemented in selected patients with inoperable meningiomas to provide additional treatment options. Finally, although Collamati et al. [39] devised a pilot study to analyze the safety and effectiveness of the [^68^Ga]Ga-DOTATOC and [^90^Y]Y-DOTATOC theranostic pair for radioguided high-grade glioma and meningioma resection, their findings still need to be externally validated before being implemented in clinical practice.

### 4.5. Ongoing Clinical Trials and Future Perspectives

Four ongoing clinical trials are currently evaluating [^68^Ga]Ga-DOTATOC and [^68^Ga]Ga-DOTATATE in patients with meningiomas [82,83], pituitary adenomas [80], and other SSTR2-positive brain tumors [81]. Three trials are focused on analyzing [^68^Ga]Ga-DOTA-SSTR diagnostic accuracy: (1) compared to MRI for meningiomas [81]; (2) for distinguishing normal pituitary tissue versus pituitary tumors [80]; (3) for measuring post-radiation tumor response [82]; and/or (4) for correlating tracer uptake to SSTR2 expression and other tumor molecular patterns [80,81,82]. Separately, the trial led by Merrell aims to evaluate the safety and effectiveness of neurotheranostics ([^68^Ga]Ga-DOTATATE and [^177^Lu]Lu-DOTATATE) for meningiomas in terms of progression-free survival, overall survival, and adverse events. While the trial conducted by Filipsson Nyström [80] is set to include patients with primary untreated pituitary adenomas, the three other trials [81,82,83] are devised to involve patients with recurrent meningiomas, planning to receive radiation, and with radiologically measurable volumes. The findings achieved with these trials are expected to provide a more comprehensive and heterogeneous understanding of the benefits of [^68^Ga]Ga-DOTA-SSTR PET studies for different populations. Future studies should also evaluate the role of intraoperative [^68^Ga]Ga-DOTA-SSTR PET-guided resection of skull base and transosseous meningiomas in terms of surgical feasibility, additional operating time, the extent of tumor resection, and its impact on postoperative patient performance status.

### 4.6. Limitations

Our review has some limitations. All included studies were retrospective case reports and case series likely exposed to selection bias. Owing to the recent introduction of [^68^Ga]Ga-DOTA-SSTR in clinical settings and the reduced availability of studies currently published, we have also included many case reports. These case reports may limit any statistical evaluation of the accuracy, sensitivity, and specificity of this technique as of the current day, but offer valuable information on the several potential uses of [^68^Ga]Ga-DOTA-SSTR in neuro-oncology. The high costs and recent development of [^68^Ga]Ga-DOTA-SSTR tracers may have prevented the implementation of such a technique worldwide, limiting published studies and our findings only to experiences from a few selected institutions. Due to a lack of granular data, we could neither comprehensively assess differences in SUVmax rates among different tumors nor the impact of [^68^Ga]Ga-DOTA-SSTR PET studies on post-treatment patient outcomes. Future studies should better analyze the diagnostic accuracy of [^68^Ga]Ga-DOTA-SSTR PET imaging compared to other imaging modalities for each type of brain tumor and how these studies impact the management of affected patients.

## 5. Conclusions

The recent development of [^68^Ga]Ga-DOTA-SSTR PET tracers in brain tumors has provided a valuable diagnostic adjunct primarily in the management of patients with meningiomas and pituitary adenomas/carcinomas. In particular, current routine applications of [^68^Ga]Ga-DOTA-SSTR PET imaging are shown to correlate with improved diagnostic accuracy, delineation of radiotherapy targets, and patient eligibility for radionuclide therapies. Ongoing trials are set to better define the diagnostic performance of these approaches, and future studies should evaluate the impact of [^68^Ga]Ga-DOTA-SSTR PET studies for imaging-guided surgical tumor resections.

## Figures and Tables

**Table 1:** Summary of all included studies and pooled patients.

Characteristics	Total	Diagnostic Purposes	Treatment Planning	Neuro-Theranostics
Cohort size, No. (%)				
Patients	1030	644 (62.5%)	337 (32.7%)	49 (4.8%)
Lesions	1277	867 (67.9%)	356 (27.9%)	54 (4.2%)
Pathology, No. lesions (%)				
Meningioma	1196 (93.6%)	806 (93%)	355 (99.7%)	40 (74.1%)
Pituitary adenoma	36 (2.8%)	36 (4.2%)	0 (0%)	0 (0%)
Brain metastases	15 (1.2%)	15 (1.7%)	0 (0%)	0 (0%)
High-grade glioma	12 (0.9%)	0 (0%)	0 (0%)	12 (22.2%)
Pituitary carcinoma	6 (0.5%)	3 (0.3%)	1 (0.3%)	2 (3.7%)
Granulomatous inflammation	2 (0.2%)	2 (0.2%)	0 (0%)	0 (0%)
Lymphoma	2 (0.2%)	2 (0.2%)	0 (0%)	0 (0%)
Glomus jugulare	1 (0.1%)	1 (0.1%)	0 (0%)	0 (0%)
Hemangioma	1 (0.1%)	1 (0.1%)	0 (0%)	0 (0%)
Schwannoma	1 (0.1%)	1 (0.1%)	0 (0%)	0 (0%)
Tracer, No. patients (%)				
DOTATOC	548 (53.2%)	275 (42.7%)	250 (74.2%)	23 (46.9%)
DOTATATE	403 (39.1%)	329 (51.1%)	68 (20.2%)	6 (12.2%)
DOTANOC	59 (5.7%)	40 (6.2%)	19 (5.6%)	0 (0%)
DOTATOC/DOTATATE	20 (1.9%)	0 (0%)	0 (0%)	20 (40.8%)

**Table 2:** Overview of all included studies using [^68^Ga]Ga-DOTA-SSTR PET for diagnostic purposes.

Authors—Year	Reason for [^68^Ga]Ga-PET	Patients/ Lesions	Pathology/Tracer	MBq Dose/ SUVmax	Findings
Henze, 2001 [79]	Diagnostic feasibility	3/ 8	Meningioma/ DOTATOC	175/ 10.6	Higher accuracy in evaluating intraosseous extent at skull base
Afshar-Oromieh, 2012 [30]	Compare the accuracy to MRI	134/ 190	Meningioma/ DOTATOC	139.6 (55–307)/ 4.6	Higher sensitivity for tumors at falx cerebri and skull base
Graf, 2012 [31]	Detect infracranial invasion	16/ 16	Meningioma/ DOTATOC	70–120/ N/A	Higher accuracy in evaluating infracranial invasion
Law, 2013 [34]	Systemic tumor staging	1/ 1	Meningioma/ DOTATATE	250/ N/A	Incidental meningioma detection
Yilmaz, 2013 [35]	Diagnostic confirmation	1/ 1	Meningioma/ DOTATOC	N/A/ N/A	High DOTATOC uptake with low FDG uptake
Boss, 2014 [37]	Assess the accuracy of hybrid PET/MRI	3/ 3	Meningioma/ DOTATATE	135 (126–153)/ N/A	Similar accuracy to PET/CT
Slotty, 2014 [38]	Differentiate recurrence from scarring	1/ 1	Meningioma/ DOTATATE	N/A/ N/A	Higher accuracy in differentiating tumor (uptake) versus scar (no uptake)
Zhao, 2014 [16]	Assess pituitary reserve and residual adenoma	35/ 35	Pituitary adenoma/ DOTATATE	100–200/ Residual 3.6 Reserve 5.8	Higher DOTATOC uptake pituitary reserve and higher FDG uptake residual tumor
Afshar-Oromieh, 2015 [13]	Assess the accuracy of hybrid PET/MRI	15/ 33	Meningioma/ DOTATOC	190 (100–265)/ PET/CT 10.5 PET/MRI 8.3	High sensitivity/specificity (PET) and morphological evaluation (MRI)
Klingenstein, 2015 [40]	Differential diagnosis optic pathway lesions	13/ 13	Meningioma (10), other (3)/DOTATATE	N/A/ N/A	Confirm meningioma (high uptake) vs. other diseases (no/low uptake)
Rachinger, 2015 [14]	Differentiate tumor from tumor-free tissue	21/ 21	Meningioma/ DOTATATE	150/ Threshold 2.3	SUVmax 2.3 threshold to differentiate tumor tissue versus non-tumor tissue
Taneja, 2015 [42]	Diagnostic confirmation	1/ 1	Granulomatous inflammation/ DOTATATE	N/A/ N/A	High uptake in granulomatous inflammation
Xiao, 2015 [43]	Diagnostic confirmation	1/ 5	Pituitary carcinoma/ DOTATATE	N/A/ N/A	High expression SSTR2 in pituitary carcinoma plus FDG-undetected lesions (4)
Basu, 2016 [44]	Diagnostic confirmation	1/ 2	Pituitary adenoma and meningioma/ DOTATATE	N/A/ N/A	Synchronous detection meningioma plus pituitary adenoma
Sommerauer, 2016 [45]	Predict tumor growth rate	23/ 64	Meningioma/ DOTATATE	150/ Intracranial 11.2 Transosseous 43.3	DOTATATE-PET predicts tumor growth in grade-1 and grade-2 meningiomas
Garmes, 2016 [47]	Diagnostic confirmation	1/ 1	Pituitary carcinoma/ DOTATATE	N/A/ N/A	Confirmation of pituitary carcinoma with metastases
Kunz, 2017 [15]	Detect intraosseous invasion	82/ 82	Meningioma/ DOTATATE	150/ Extraosseous 7.6 Transosseous 14.2	Higher detection transosseous extent of meningiomas than contrast-enhanced MRI
Unterrainer, 2017 [48]	Diagnostic confirmation	1/ 3	Brain metastasis/ DOTATATE	N/A/ N/A	High tracer uptake in metastases from thyroid carcinoma
Villanueva-Meyer, 2018 [50]	Tumor staging	1/ 5	Meningioma/ DOTATATE	136.9/ N/A	Detection of multiple dural-based plus liver metastases
Dressen, 2019 [52]	Assess SSTR2 expression	67/ 67	Meningioma/ DOTATATE	N/A/ 11.2	High DOTATATE uptake (SSTR2 expression) may coincide with low FDG uptake (low metabolic rate)
Ivanidze, 2019 [53]	Diagnostic confirmation	20/ 49	Meningioma/ DOTATATE	N/A/ 23.3	Differentiate residual meningioma versus post-therapy change (target lesion/superior sagittal sinus)
Purandare, 2019 [54]	Differential diagnosis	40/ 40	Meningioma (31), dural metastases (4)/DOTATOC	N/A/ Meningioma 12.7 Metastases 6.0	Higher accuracy in differentiating meningiomas (high uptake) from dural-based metastasis (low uptake)
Unterrainer, 2019 [55]	Diagnostic confirmation and differential	1/ 2	Meningioma and dural metastasis/ DOTATOC	N/A/ Meningioma 88 Metastases 2.9	Differentiation of concurrent meningioma and dural-based metastasis
Unterrainer, 2019 [56]	Diagnostic confirmation	1/ 1	Meningioma/ DOTATATE	N/A/ N/A	Detection of multiple meningiomas and liver, lung, and bone metastases
Bashir, 2020 [58]	Differential diagnosis	2/ 2	Meningioma (1), lymphoma (1)/ DOTATOC	N/A/ Meningioma 11.5 Lymphoma 11.8	Different lesions showed similar SUVmax
Bashir, 2020 [59]	Relation tracer uptake and SSRT2 expression	15/ 15	Meningioma/ DOTATOC	105/ 20.9 (8.4–102)	Tumor-to-background ratio best PET metric for the evaluation of SSTR2 expression
Ueberschaer, 2020 [60]	Assess the extent of resection	49/ 52	Meningioma/ DOTATATE	150/ 4.2 (2.8–13.9)	Postoperative PET has higher accuracy than Simpson grade
Assadi, 2021 [61]	Assess radionuclide response	1/ 1	Meningioma/ DOTATOC	N/A/ Pre 11.76 Post 9.02	Reduced SUVmax after radionuclide
Barone, 2021 [62]	Assess SUV changes after radiosurgery	20/ 20	Meningioma/ DOTATOC	N/A/ Pre 20.8 (2.3–52) Post 12.5 (2.3–44)	Post-radiosurgery reduction of SUVmax in 7/12 patients
Einhelling, 2021 [63]	Compare the accuracy to MRI	57/ 112	Meningioma/ DOTATOC	163.2/ 10.8	High sensitivity in detecting small sphenoidal or orbital meningiomas
Fabritius, 2021 [64]	Differential diagnosis	1/ 1	Brain metastasis/ DOTATOC	N/A/ N/A	High expression SSTR2 in breast cancer metastasis
Hanslund-Vinding, 2021 [65]	Assess residual tumor post-surgery	6/ 6	Meningioma/ DOTATOC	N/A/ N/A	DOTATATE plus biopsy “area of doubt” to confirm complete tumor resection
Kaya, 2021 [67]	Diagnostic confirmation	1/ 5	Pituitary carcinoma/ DOTATATE	N/A/ N/A	Confirmation of spinal cord metastases
Ragni, 2021 [70]	Diagnostic confirmation	1/ 1	Brain metastasis/ DOTATOC	N/A/ N/A	Incidental finding pituitary mass in NEN follow-up
Vay, 2021 [71]	Diagnostic confirmation	1/ 1	Meningioma/ DOTATATE	N/A/ N/A	Confirm optic nerve sheath meningioma
Yarmohammadi, 2021 [72]	Diagnostic confirmation	1/ 1	Meningioma/ DOTATATE	N/A/ 2.4	Confirm optic nerve sheath meningioma
Balabanc Genc, 2022 [73]	Diagnostic confirmation	1/ 1	Glomus jugulare/ DOTATATE	N/A/ N/A	Confirm recurrent glomus jugulare invading the cerebellum
Farce, 2022 [74]	Diagnostic confirmation	1/ 1	Schwannoma/DOTATOC	N/A/ N/A	Low DOTATOC uptake, low FDG uptake, high choline uptake
Filizoglu, 2022 [75]	Diagnostic confirmation	1/ 1	Pituitary carcinoma/ DOTATATE	N/A/ N/A	Confirm pituitary carcinoma plus systemic metastases
Fine, 2022 [76]	Diagnostic confirmation	1/ 1	Brain metastasis/ DOTATATE	N/A/ N/A	Confirm pineal metastasis in neuroendocrine neoplasm follow-up
Mairal, 2022 [77]	Diagnostic confirmation	1/ 1	Meningioma/ DOTATOC	N/A/ N/A	Mismatch low DOTATOC and high FDG
Saidi, 2022 [78]	Diagnostic confirmation	1/ 1	Hemangioma/DOTATATE	N/A/ 39.8	High DOTATATE uptake post-traumatic hemangioma

**Table 3:** Overview of all included studies using ^68^Ga-DOTA-SSTR PET for planning radiotherapy and/or surgical resection.

Authors—Year	Reason for [^68^Ga]Ga-PET	Patients/ Lesions	Pathology/Tracer	MBq Dose/ SUVmax	Findings
Milker-Zabel, 2006 [26]	Volume contouring for fractionated SRT	26/ 27	Meningioma/ DOTATOC	156/ N/A	Change target definition in 19 patients (73%)
Gehler, 2009 [27]	Volume contouring for intensity-modulated RT	26/ 26	Meningioma/ DOTATOC	150/ N/A	Improved target definition for skull base and recurrent tumors
Nyuyki, 2010 [28]	Volume contouring for SRT	42/ 51	Meningioma/ DOTATOC	70–120/ N/A	Improved target definition for osseous infiltrated tumors
Thorwart, 2011 [29]	Volume contouring for intensity-modulated RT	1/ 1	Meningioma/ DOTATOC	N/A N/A	Lower target definition with PET/MRI than PET/CT + MRI
Combs, 2013 [32]	Volume contouring for proton/carbon therapy	70/ 70	Meningioma/ DOTATOC	N/A N/A	Improved target definition for post-resection lesions
Graf, 2013 [33]	Volume contouring for fractionated SRT	54/ 56	Meningioma/ DOTATOC	70–120/ N/A	Improved target definition for skull base tumors (lower GTV)
d’Amico, 2014 [36]	Volume contouring for cyber knife	1/ 1	Pituitary carcinoma/ DOTATOC	N/A N/A	Improved target definition for pituitary carcinoma invading the cavernous sinus
Maclean, 2017 [46]	Volume contouring for RT	10/ 10	Meningioma/ DOTATATE	100/ N/A	Improved inter-observer variability in contouring
Stade, 2018 [49]	Volume contouring for RT	10/ 10	Meningioma/ DOTATATE	N/A N/A	Reduction in treatment volumes and doses to organs at risk
Zollner, 2018 [51]	Volume contouring for RT	20/ 20	Meningioma/ DOTATATE	N/A/ 9.76	Extended safety margins (>1 cm) favor higher local control
Acker, 2019 [17]	Volume contouring for cyber knife	10/ 11	Meningioma/ DOTATOC	165/ N/A	PET/MRI improves target definition in training doctors
Graef, 2021 [18]	Volume contouring for cyber knife	8/ 10	Meningioma/ DOTATOC	168/ 5.6	Improved target definition for optic nerve sheath meningiomas
Guinto-Nishimura, 2021 [19]	Intraoperative resection guidance	1/ 1	Meningioma/ DOTATOC	N/A N/A	Improved target definition for intraosseous meningioma
Kowalski, 2021 [66]	Volume contouring for RT	19/ 19	Meningioma/ DOTATATE	N/A/ 61.2	Improved target definition for intraosseous and falx meningiomas
Mahase, 2021 [20]	Volume contouring for hypofractionated RT	8/ 9	Meningioma/ DOTATATE	N/A N/A	Reduction in treatment volumes and doses to organs at risk
Pelak, 2021 [69]	Volume contouring for proton therapy	30/ 34	Meningioma/ DOTANOC DOTATOC DOTATATE	75-273/ 13.5 (6.6–31.2)	Identification of MRI-undetected lesions and improved definition of intraosseous extension

**Table 4:** Overview of all included studies using [^68^Ga]Ga-DOTA PET for planning neurotheranostics therapy.

Authors—Year	Reason for [^68^Ga]Ga-PET	Patients/ Lesions	Pathology/Tracer	MBq Dose/ SUVmax	Findings
Collamati, 2015 [39]	Estimate uptake of [^90^Y]Y-DOTATOC tracer	23/ 23	High-grade glioma (12), meningioma (11)/ DOTATOC	N/A/ N/A	Pilot study using ^68^Ga-DOTATOC to estimate uptake of ^90^Y-DOTATOC radiotracer to guide tumor resection
Novruzov, 2015 [41]	Assess SSTR2 expression for [^177^Lu]Lu-DOTATE therapy	1/ 1	Pituitary carcinoma/ DOTATATE	N/A/ Pre 6.8 Post 4.7	High [^68^Ga]Ga -DOTATATE uptake (SSTR2 expression) in pituitary carcinoma with use for [^177^Lu]Lu-DOTATE therapy
Seystahl, 2016 [21]	Assess SSTR2 expression for [^177^Lu]Lu-DOTATE therapy	20/ 20	Meningioma/ DOTATOC, DOTATATE	N/A/ N/A	Higher SSTR2 expression in a tumor correlated with higher rates of stable disease at 6 months post-therapy
Verburg, 2019 [57]	Intraarterial injection versus venous infusion to evaluate tracer uptake for [^177^Lu]Lu-DOTATE therapy	4/ 9	Meningioma/ DOTATATE	N/A/ Intraarterial 21.6 Venous 7.1	Selective intraarterial DOTATATE injection increases tracer uptake in meningiomas with insufficient venous uptake confirming eligibility for [^177^Lu]Lu-DOTATE therapy
Lybik, 2021 [68]	Assess SSTR2 expression for [^177^Lu]Lu-DOTATE therapy	1/ 1	Pituitary carcinoma/ DOTATATE	N/A/ 24	High [^68^Ga]Ga-DOTATATE uptake (SSTR2 expression) in pituitary carcinoma with use for [^177^Lu]Lu-DOTATE therapy

**Table 5:** Overview of all ongoing clinical trials on [^68^Ga]Ga-DOTA-SSTR PET in neuro-oncology.

Investigator—Trial Number	Enrollment/ Pathology	Inclusion Criteria	Exclusion Criteria	Primary Outcomes	Secondary Outcomes
Filipsson Nyström NCT02419664 [80]	22/ Pituitary adenomas	(1) Naïve, unoperated pituitary tumor with GH or ACTH or TSH production or non-functioning without treatment with somatostatin analogues or dopamine agonists.	(1) Pregnancy or lactating; (2) Isolated prolactin-producing tumors; (3) Overproduction of gonadotrophins; (4) Carcinoids (ectopic CRF production); (5) Allergy to [^68^Ga]Ga-DOTATOC.	SUVmax in pituitary tumors compared to normal pituitary	(1) Correlate [^68^Ga]Ga-DOTATOC uptake with expression of SSTR2; (2) Adverse events; (3) Detection of post-surgery tumor recurrence.
Ivanidze NCT04081701 [81]	90/ SSTR-positive brain tumors	(1) Age ≥ 18 years; (2a) Meningioma diagnosis at pathology and suspected recurrence or residual disease at MRI; OR (2b) SSTR2-positive brain tumors; (3) 1.5T or 3T MRI as per clinical standard-of-care and [^68^Ga]Ga-DOTATATE PET/CT with the PET portion fused with MRI.	(1) Contraindications to gadolinium-based contrast agent; (2) Allergy to [^68^Ga]Ga-DOTATATE; (3) Pregnancy.	Diagnostic accuracy of [^68^Ga]Ga-DOTATATE PET/MRI will be compared to MRI alone.	Correlate [^68^Ga]Ga-DOTATATE PET/MRI with expression of: (1) SSTR2; (2) Ki67; (3) Progesterone receptor; (4) EGFR.
Johnson NCT03953131 [82]	12/ Meningioma	(1) Any meningioma with ≥10 mm measurable residual disease; (2) Planned radiation therapy; (3) Willing and able to give consent and participate in all evaluations.	(1) Neurofibromatosis type 1 or 2; (2) Pregnancy (3) Contraindication to MRI; (4) Body weight greater than 400 pounds (lbs.) (181.4 kg).	(1) Diagnostic accuracy of [^68^Ga]Ga-DOTATATE PET/CT; (2) Metabolic response to radiation measured by reduction in tumor to background ratio (SUVmax of tumor compared to background parenchyma).	N/A
Merrell NCT04082520 [83]	41/ Meningioma	(1) Prior treatment (surgery and/or radiation); (2) Radiographic evidence of progression; (3) Measurable disease; (4) Prior fractionated or stereotactic radiation at the site of progressive meningioma; (5) ECOG-PS ≤ 2; (6) ANC ≥ 1500/mm; (7) PLT ≥ 100,000/mm; (8) Hb ≥ 9 g/dL; (9) Direct bilirubin < 1.5 × ULN; (10) AST ≤ 3 × ULN; (11) PT/INR/PTT ≤ 1.5 × ULN; (12) CCL ≤ 40 mL/mm; (13) Negative pregnancy test; (14) AST ≤ 3 × ULN; (15) Willing and able to give consent and participate in all evaluations.	(1) Eligibility for surgery or radiation with curative intent; (2) Pregnancy, nursing, or childbearing age; (3) Ineligible due to co-morbid systemic illnesses or immunocompromised; (4) Contraindication to MRI; (5) Other active malignancy ≤ 2 years prior to registration; (6) Myocardial infarction ≤ 6 months or congestive heart failure; (7) Spontaneous urinary incontinence; (8) Significant toxicity related to previous radiation; (9) Optic nerve sheath meningioma, extracranial meningioma.	Progression-free survival at 6 months after [^68^Ga]Ga-DOTATATE PET/MRI + [^177^Lu]Lu-DOTATATE therapy.	(1) Overall survival; (2) Progression free survival (up to 5 years); (3) Adverse events.

## Supplementary Materials

The following supporting information can be downloaded at: https://www.mdpi.com/article/10.3390/cancers14122925/s1, Supplementary File S1: Risk of bias assessments for all included studies.
