# Supplementary material for: The Role of [68Ga]Ga-DOTA-SSTR PET Radiotracers in Brain Tumors: A Systematic Review of the Literature and Ongoing Clinical Trials
Source: Cancers (Basel). 2022 Jun 14;14(12):2925. doi: 10.3390/cancers14122925 (PMC9221214; doi:10.3390/cancers14122925)
Supplement: Supplementary file 1 [file cancers-14-02925-s001.zip › Supplementary File S1 - Risk of bias assessment for included studies.pdf]

Supplementary File S1. Risk of bias assessments for included studies

**Joanna Briggs Institute Checklist for Case Reports – Criteria**
1. Were patient's demographic characteristics clearly described?
2. Was the patient's history clearly described and presented as a timeline?
3. Was the current clinical condition of the patient on presentation clearly described?
4. Were diagnostic tests or assessment methods and the results clearly described?
5. Was the intervention(s) or treatment procedure(s) clearly described?
6. Was the post-intervention clinical condition clearly described?
7. Were adverse events (harms) or unanticipated events identified and described?
8. Does the case report provide takeaway lessons?
**Responses Options**: Yes, No, Unclear, Not Applicable (NA)
**Quality Rating**: Poor 0 – 2; Fair 3 – 5; Good 6 – 8

Study	1	2	3	4	5	6	7	8	Rating
Thorwarth et al. – 2011 [29]	Yes	Yes	Yes	Yes	Yes	Yes	Yes	Yes	8 – Good
Law et al. – 2013 [34]	Yes	Yes	Yes	Yes	Yes	Yes	Yes	Yes	8 – Good
Yilmaz et al. – 2013 [35]	Yes	Yes	Yes	Yes	Yes	Yes	Yes	Yes	8 – Good
d'Amico et al. – 2014 [36]	Yes	Yes	Yes	Yes	Yes	Yes	Yes	Yes	8 – Good
Slotty et al. – 2014 [38]	Yes	Yes	Yes	Yes	Yes	Yes	Yes	Yes	8 – Good
Novruzov et al. – 2015 [41]	Yes	Yes	Yes	Yes	Yes	Yes	Yes	Yes	8 – Good
Taneja et al. – 2015 [42]	Yes	Yes	Yes	Yes	Yes	Yes	Yes	Yes	8 – Good
Basu et al. – 2016 [44]	Yes	Yes	Yes	Yes	Yes	Yes	Yes	Yes	8 – Good
Unterrainer et al. – 2017 [48]	Yes	Yes	Yes	Yes	Yes	Yes	Yes	Yes	8 – Good
Villanueva-Meyer et al. – 2018 [50]	Yes	Yes	Yes	Yes	Yes	Yes	Yes	Yes	8 – Good
Dressen et al. – 2019 [52]	Yes	Yes	Yes	Yes	Yes	Yes	Yes	Yes	8 – Good
Unterrainer et al. – 2019 [55]	Yes	Yes	Yes	Yes	Yes	Yes	Yes	Yes	8 – Good
Unterrainer et al. – 2019 [56]	Yes	Yes	Yes	Yes	Yes	Yes	Yes	Yes	8 – Good
Assadi et al. – 2021 [61]	Yes	Yes	Yes	Yes	Yes	Yes	Yes	Yes	8 – Good
Fabritius et al. – 2021 [64]	Yes	Yes	Yes	Yes	Yes	Yes	Yes	Yes	8 – Good
Guinto-Nishimura et al. – 2021 [19]	Yes	Yes	Yes	Yes	Yes	Yes	Yes	Yes	8 – Good
Kaya et al. – 2021 [67]	Yes	Yes	Yes	Yes	Yes	Yes	Yes	Yes	8 – Good
Lybik et al. – 2021 [68]	Yes	Yes	Yes	Yes	Yes	Yes	Yes	Yes	8 – Good
Ragni et al. – 2021 [70]	Yes	Yes	Yes	Yes	Yes	Yes	Yes	Yes	8 – Good
Vay et al. – 2021 [71]	Yes	Yes	Yes	Yes	Yes	Yes	Yes	Yes	8 – Good
Yarmohammadi et al. – 2021 [72]	Yes	Yes	Yes	Yes	Yes	Yes	Yes	Yes	8 – Good
Balaban Genc et al. – 2022 [73]	Yes	Yes	Yes	Yes	Yes	Yes	Yes	Yes	8 – Good
Farce et al. – 2022 [74]	Yes	Yes	Yes	Yes	Yes	Yes	Yes	Yes	8 – Good
Filizoglu et al. – 2022 [75]	Yes	Yes	Yes	Yes	Yes	Yes	Yes	Yes	8 – Good
Fine et al. – 2022 [76]	Yes	Yes	Yes	Yes	Yes	Yes	Yes	Yes	8 – Good
Mairal et al. – 2022 [77]	Yes	Yes	Yes	Yes	Yes	Yes	Yes	Yes	8 – Good

Saidi et al. – 2022 [78]	Yes	Yes	Yes	Yes	Yes	Yes	Yes	Yes	8 – Good

Joanna Briggs Institute Checklist for Case Series – Criteria
1. Were there clear criteria for inclusion in the case series?
2. Was the condition measured in a standard, reliable way for all participants included in the case series?
3. Were valid methods used for identification of the condition for all participants included in the case series?
4. Did the case series have consecutive inclusion of participants?
5. Did the case series have complete inclusion of participants?
6. Was there clear reporting of the demographics of the participants in the study?
7. Was there clear reporting of clinical information of the participants?
8. Were the outcomes or follow up results of cases clearly reported?
9. Was there clear reporting of the presenting site(s)/clinic(s) demographic information?
10. Was statistical analysis appropriate?
**Responses Options**: Yes, No, Unclear, Not Applicable (NA)
**Quality Rating**: Poor 0 – 3; Fair 4 – 7; Good 8 – 10

Study	1	2	3	4	5	6	7	8	9	10	Rating
Henze et al. – 2001 [79]	Yes	Yes	Yes	Yes	Yes	Yes	Yes	Yes	No	NA	8 – Good
Milker-Zabel et al. – 2006 [26]	Yes	Yes	Yes	Yes	Yes	Yes	Yes	Yes	No	NA	8 – Good
Gehler et al. – 2009 [27]	Yes	Yes	Yes	Yes	Yes	Yes	Yes	Yes	No	NA	8 – Good
Nyuyki et al. – 2010 [28]	Yes	Yes	Yes	Yes	Yes	Yes	Yes	Yes	No	Yes	9 – Good
Afshar-Oromieh et al. – 2012 [30]	Yes	Yes	Yes	Yes	Yes	Yes	Yes	Yes	No	NA	8 – Good
Graf et al. – 2012 [31]	Yes	Yes	Yes	Yes	Yes	Yes	Yes	Yes	No	Yes	9 – Good
Combs et al. – 2013 [32]	Yes	Yes	Yes	Yes	Yes	Yes	Yes	Yes	No	NA	8 – Good
Graf et al. – 2013 [33]	Yes	Yes	Yes	Yes	Yes	Yes	Yes	Yes	No	Yes	9 – Good
Boss et al. – 2014 [37]	Yes	Yes	Yes	Yes	Yes	Yes	Yes	Yes	No	NA	8 – Good
Zhao et al. – 2014 [16]	Yes	Yes	Yes	Yes	Yes	Yes	Yes	Yes	No	Yes	9 – Good
Afshar-Oromieh et al. – 2015 [13]	Yes	Yes	Yes	Yes	Yes	Yes	Yes	Yes	Yes	Yes	10 – Good
Collamati et al. – 2015 [39]	Yes	Yes	Yes	Yes	Yes	Yes	Yes	Yes	No	Yes	9 – Good
Klingenstein et al. – 2015 [40]	Yes	Yes	Yes	Yes	Yes	Yes	Yes	Yes	Yes	NA	9 – Good
Rachinger et al. – 2015 [14]	Yes	Yes	Yes	Yes	Yes	Yes	Yes	Yes	No	NA	8 – Good
Seystahl et al. – 2016 [21]	Yes	Yes	Yes	Yes	Yes	Yes	Yes	Yes	Yes	Yes	10 – Good
Sommerauer et al. – 2017 [45]	Yes	Yes	Yes	Yes	Yes	Yes	Yes	Yes	No	Yes	9 – Good
Kunz et al. – 2017 [15]	Yes	Yes	Yes	Yes	Yes	Yes	Yes	Yes	Yes	Yes	10 – Good
Maclean et al. – 2017 [46]	Yes	Yes	Yes	Yes	Yes	Yes	Yes	Yes	No	NA	8 – Good
Stade et al. – 2018 [49]	Yes	Yes	Yes	Yes	Yes	Yes	Yes	Yes	Yes	Yes	10 – Good
Zollner et al. – 2018 [51]	Yes	Yes	Yes	Yes	Yes	Yes	Yes	Yes	Yes	Yes	10 – Good
Acker et al. – 2019 [17]	Yes	Yes	Yes	Yes	Yes	Yes	Yes	Yes	Yes	Yes	10 – Good
Ivanidze et al. – 2019 [53]	Yes	Yes	Yes	Yes	Yes	Yes	Yes	Yes	No	Yes	9 – Good
Purandare et al. – 2019 [54]	Yes	Yes	Yes	Yes	Yes	Yes	Yes	Yes	No	NA	8 – Good
Verburg et al. – 2019 [57]	Yes	Yes	Yes	Yes	Yes	Yes	Yes	Yes	No	NA	8 – Good
Bashir et al. – 2020 [58]	Yes	Yes	Yes	Yes	Yes	Yes	Yes	Yes	No	NA	8 – Good
Bashir et al. – 2020 [59]	Yes	Yes	Yes	Yes	Yes	Yes	Yes	Yes	No	NA	8 – Good
Ueberschaer et al. – 2020 [60]	Yes	Yes	Yes	Yes	Yes	Yes	Yes	Yes	No	Yes	9 – Good

Barone et al. – 2021 [62]	Yes	Yes	Yes	Yes	Yes	Yes	Yes	Yes	No	Yes	9 – Good
Einhelling et al. – 2021 [63]	Yes	Yes	Yes	Yes	Yes	Yes	Yes	Yes	Yes	Yes	10 – Good
Graef et al. – 2021 [18]	Yes	Yes	Yes	Yes	Yes	Yes	Yes	Yes	Yes	Yes	10 – Good
Haslund-Vinding et al. – 2021 [65]	Yes	Yes	Yes	Yes	Yes	Yes	Yes	Yes	No	NA	8 – Good
Kowalski et al. – 2021 [66]	Yes	Yes	Yes	Yes	Yes	Yes	Yes	Yes	No	Yes	9 – Good
Mahase et al. – 2021 [20]	Yes	Yes	Yes	Yes	Yes	Yes	Yes	Yes	Yes	Yes	10 – Good
Pelak et al. – 2021 [69]	Yes	Yes	Yes	Yes	Yes	Yes	Yes	Yes	Yes	Yes	10 – Good
